# Inverted Toe Nail: A New Remedy

**Published:** 1851-02

**Authors:** Benj. P. Drake

**Affiliations:** Lexington, Ky.


Art. II.—*Inverted Toe Nail.* A new Remedy. By Benj. P. Drake, M. D., of Lexington, Ky.

There are but few diseases which inflict on the patient a greater amount of suffering, and on the medical attendant a greater amount of mortification, than the one that stands at the head of this article. The exquisitely sensible granulations in which the nail is embedded, frequently produce such intense pain as entirely to prevent the patient from wearing a shoe and from walking. Besides this, the obstinacy of the malady frequently compels the practitioner to resort, one after another, to all the various remedies which have been suggested for its removal, almost every one of which is painful, some of them excruciatingly so, and at last, mortified and foiled, he gives up the case without having accomplished a cure.

The treatment pursued in the following case is worthy the attention of the faculty and a fair trial of its efficacy, from the consideration that it is without pain in its application and that in this instance at least it has been successful:

Case. Miss A. E. T., æt. about 16, of irritable constitution, has been for the last three years the subject of inverted toe nail in the great toes of both feet. Almost every remedy that has been suggested for its relief had been resorted to, but without success. The nail had been scraped very thin, caustic had been extensively and frequently applied, the roller had been worn for months, and lastly, the diseased sides of the nails had been split down to the glands, and then with the forceps, torn out by the roots. The relief following these severe measures was but partial and temporary, and in the course of a few weeks or months the disease was as distressing and painful as ever.

In this state of the case it occurred to me to make trial of the common tannic acid, which I applied in the following manner: I placed a portion of the tannin in its dry state on the nail, and then raising the exuberant granulations, with the tortoise shell handle of a lancet, I gently but carefully pressed it down to the embedded edge of the nail and filled the cavity with it, after which I covered the whole of the proud flesh with the same application, and enveloped the toe with a roller bandage. As no pain followed, the dressing was permitted to remain until the fourth day. On its removal, the improvement was so manifest that I had no hesitation in using the remedy again, which I did precisely as at first. To be brief, six or eight applications of the tannin, at intervals of three or four days, were made, and the cure appears to be complete and perfect.

